# 2SLOD-HCG: HCG Test Strip Concentration Prediction Network

**DOI:** 10.3390/s25175378

**Published:** 2025-09-01

**Authors:** Qi Hu, Jinshu Zhao, Shimin Kan, Qiang Shi, Ning Wang, Jiajian Li, Zhifang Ma

**Affiliations:** 1 School of Artificial Intelligence, Changchun University of Science and Technology, Changchun 130022, China; 2023102067@mails.cust.edu.cn (S.K.);; 2 School of Electronic Information Engineering, Changchun University of Science and Technology, Changchun 130022, China; 2023100778@mails.cust.edu.cn (J.Z.); 2023100718@mails.cust.edu.cn (N.W.); 3 State Key Laboratory of Polymer Physics and Chemistry, Changchun Institute of Applied Chemistry, Changchun 130022, China; shiqiang@ciac.ac.cn (Q.S.); zfma@ciac.ac.cn (Z.M.)

**Keywords:** concentration detection, multi-scale fusion, lightweight attention mechanism, 2SLOD-HCG

## Abstract

Human chorionic gonadotropin (HCG) is an essential biomarker for the evaluation and diagnosis of early pregnancy, multiple pregnancies, and ectopic pregnancies. However, the accuracy of test strip interpretation is often compromised by inconvenient and uncomfortable professional testing, the black-box nature of AI-based detection methods, and variations in image quality caused by mobile photography and lighting conditions. To address these challenges, we propose 2SLOD-HCG, a novel network for test strip concentration detection. Our approach introduces an enhanced spatial pyramid pooling (SPP) module to better integrate multi-scale receptive field information and incorporates an elastic variational cross-FPN structure augmented with lightweight transformer blocks to strengthen global feature perception. Furthermore, a SimAM attention mechanism is applied to highlight critical local features. These improvements collectively enhance the network’s ability to capture both fine-grained and global contextual information. We constructed a dataset of 50,000 augmented test strip images collected under three lighting conditions and four mobile photography scenarios. The results demonstrate that 2SLOD-HCG achieves superior accuracy and robustness compared to existing YOLO-based baselines, particularly in detecting the small color-developing regions of test strips.

## 1. Introduction

Human chorionic gonadotropin (HCG) is a hormone secreted by the placenta that plays a key role in supporting pregnancy and fetal development [1,2]. Quantitative detection of HCG levels is clinically valuable for diagnosing early pregnancy, multiple pregnancies, and ectopic pregnancies. In particular, monitoring the changes in HCG concentration over time can provide important guidance for the early identification of ectopic pregnancy.

Point-of-care testing (POCT) based on colloidal gold immunochromatographic strips has become an attractive option due to its simplicity and rapid results. However, traditional strip interpretation typically relies on specialized readers, which are expensive and often limited to specific manufacturers’ products [3]. To overcome these limitations, there is an urgent need for a more intelligent, practical, and accurate approach that can analyze test strip data conveniently in real-world settings. With the widespread use of smartphones equipped with high-resolution cameras, mobile-based strip analysis combined with image recognition offers a promising alternative, enabling real-time interpretation without professional devices.

Test strips can generally be divided into two categories according to the labeling materials: those based on fluorescent dyes and those based on visible chromogenic reagents [3]. Fluorescent test strips are highly sensitive but require specialized excitation light sources, making them costly and less accessible. In contrast, colloidal gold-based visible strips are more affordable and suitable for home use, but their color development is strongly affected by variations in illumination, imaging devices, and background conditions [4,5]. For example, differences in smartphone camera resolution or ambient lighting (e.g., cloudy or dim conditions) often lead to color inconsistencies in captured images. Although closed-box imaging devices can mitigate these effects, they are inconvenient for routine use.

**Figure 1:**
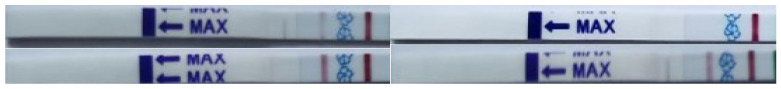
Test strip types and their appearance after reaction. The colors of the T-line and C-line after the test paper reacts and develops color. The T-line shows different colors after reacting with different concentrations.

To address these challenges, this study proposes a smartphone-based strip concentration detection algorithm inspired by the YOLO object detection framework. By improving feature extraction and enhancing robustness to environmental variations, the proposed method aims to achieve reliable, real-time interpretation of colloidal gold test strips. Figure 1 illustrates the test strips before and after a reaction, and where the intensity of the T-line color corresponds to different HCG concentrations.

## 2. Related Work

Research on medical image classification and test strip interpretation has developed rapidly with the advancement of image processing technologies. Conventional strip reading typically relies on visual observation of the color development of the test line (T-line) and control line (C-line), which is subjective and prone to error [6,7]. Although professional readers provide more accurate and quantitative results, their high cost and limited compatibility restrict widespread adoption. Recent progress in smartphone-based imaging and biosensing has shown strong potential for replacing professional readers due to their affordability, portability, and integration with intelligent algorithms [8,9,10,11,12,13].

### 2.1. Medical Testing Methods

Two mainstream approaches are currently used in strip concentration detection: visible light imaging and fluorescence imaging. Fluorescence-based systems achieve high sensitivity but generally require specialized excitation sources and closed-box imaging, which increases complexity and cost. Several studies [14,15,16,17,18,19] have proposed smartphone-compatible fluorescence systems using 3D-printed dark boxes, ultraviolet LEDs, or optical adapters. While these methods improve detection accuracy, they remain impractical for routine or home use.

In contrast, colloidal gold strips based on visible chromogenic reagents are low-cost and user-friendly. Smartphone cameras can directly capture the test results for analysis [20]. However, this approach is vulnerable to variations in ambient lighting, imaging devices, and backgrounds, leading to inconsistent color interpretation. These challenges highlight the need for more robust algorithms that can compensate for environmental variability while maintaining accuracy.

### 2.2. Current Status of Mobile Terminal Detection Applications

Smartphones have been widely applied as portable imaging and diagnostic platforms in healthcare [9,10], biosafety [11], environmental monitoring [12,13], and food safety [14,15,16]. Researchers have explored various methods to improve recognition accuracy on mobile devices, including texture-based feature extraction [21], color histogram analysis [22], and SURF-based recognition [23]. In medical applications, cloud-assisted systems have been developed for urine test strip analysis [24] and mobile disease diagnosis [25]. More recently, deep learning models such as YOLO and Darknet have been deployed on Android platforms for crop disease detection, demonstrating the feasibility of mobile AI for real-time recognition tasks [26]. These works collectively indicate that mobile platforms can support complex image recognition tasks but adapting them to medical test strips requires specialized solutions for robustness and precision.

### 2.3. Current Status of Test Line Detection

Deep learning-based object detection methods can be categorized into two groups: two-stage detectors, such as R-CNN [27], Faster R-CNN [28], and Mask R-CNN [29], which generate region proposals before classification, and one-stage detectors, such as YOLO [30,31,32] and SSD [33], which directly perform classification and regression in an end-to-end manner. One-stage methods are particularly suitable for real-time applications due to their efficiency. In addition, image segmentation approaches such as FCN [34], DeconvNet [35], and SegNet [36] have been explored for biomedical imaging tasks.

For test strip analysis, accurately detecting small color bands under varying conditions is a key challenge. Existing methods have demonstrated the feasibility of using YOLO-like architectures for this task, but their performance is limited by sensitivity to illumination, insufficient feature fusion, and lack of adaptive attention mechanisms. These limitations motivate the development of improved architectures that combine multi-scale feature fusion, global context modeling, and lightweight attention mechanisms to enhance both detection accuracy and computational efficiency.

In order to determine the number of color bands and evaluate the reliability of the test results, the primary goal of this paper is to develop an HCG test strip image enhancement method based on grayscale normalization. To do this, image preprocessing algorithms are used to precisely locate and completely enclose the color reaction region on the test strip. Next, under typical lighting conditions, we construct standard color reference spots on the test strip and determine their grayscale values. To ascertain if the image is overexposed or underexposed, these values are compared with the grayscale values at the same locations under various lighting circumstances. Depending on the results, different techniques are used to remove the impacts of lighting. In order to boost test strip feature extraction, we adjust the multi-scale feature fusion module, improve the YOLO model to increase its capacity to detect small objects, and use artificial intelligence algorithms for real-time diagnosis. The HCG concentration and its fluctuation are quantitatively determined by the system. Accurate ectopic pregnancy risk prediction is made possible by real-time diagnosis using AI systems.

While some previous studies have employed similar architectural designs, this approach achieves higher detection accuracy and generalization by incorporating multi-scale feature fusion and an adaptive attention mechanism. Compared to existing AI products on the market, our approach requires fewer computational resources and achieves faster inference speed, demonstrating its potential for practical application. While related techniques have been extensively discussed in the literature, the innovation of this study lies in improving the model structure and training strategy based on the specific characteristics of the task. The following are our primary contributions:

1. For the first time, we employ artificial intelligence to determine test strip concentrations from smartphone images. The chromogenic capacity of colloidal gold was enhanced to lower the detection limit, and AI-based techniques were integrated to accurately quantify HCG levels. This approach enables rapid detection of ectopic pregnancy using colloidal gold-based POCT test strips;
2. We suggest the S-5 module design at the bottom of the backbone network to fulfill the requirement for small-region target detection. The SimAM lightweight attention mechanism is added after the C2f module to increase detection accuracy;
3. To improve the model’s ability to perceive global characteristics, we add FPN operators to the bidirectional multi-scale fusion cross-FPN in the neck section and integrate a lightweight transformer encoder structure.

## 3. Main Research Content

In test strip concentration detection, the concentration is primarily determined by feature extraction of the T-line and C-line. To address the challenge of small object detection, this study enhances detection accuracy by introducing an improved spatial pyramid pooling (SPP) module at the terminal stage of the YOLOv8 backbone. In addition, feature pyramid network (FPN) operators are incorporated into the bidirectional multi-scale fusion cross-FPN of the neck section, while a lightweight transformer encoder is embedded to strengthen global feature representation. To further improve robustness against image degradation caused by lighting or focus variations during smartphone photography, the SimAM attention mechanism is integrated into the backbone, enabling the model to emphasize the color-developing regions of test strips.

**Figure 2:**
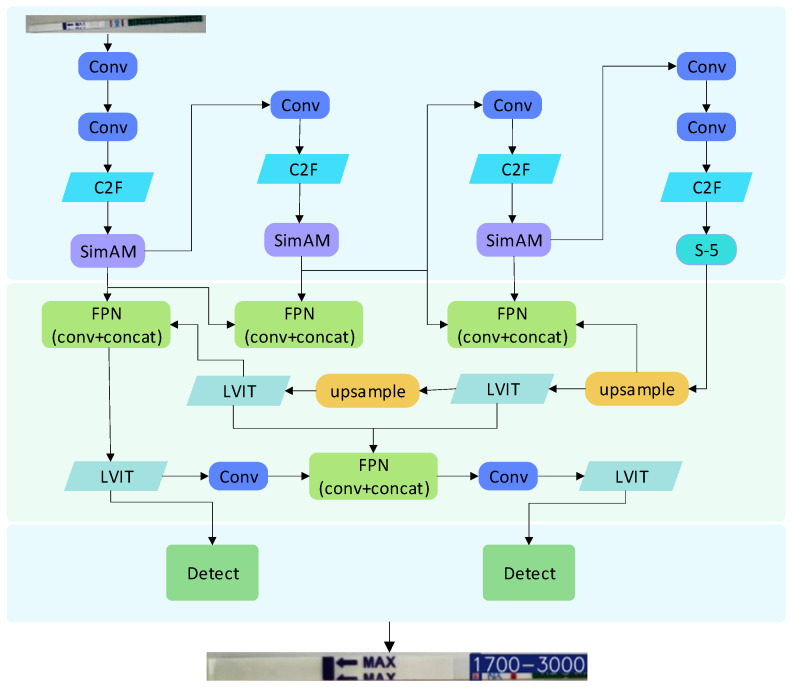
2SLOD–HCG model architecture. This figure shows the backbone network, multi–scale feature fusion, and adaptive attention modules, with feature flow and module sizes annotated.

The proposed framework, termed 2SLOD-HCG, is illustrated in Figure 2 and consists of three principal components: backbone, neck, and head. While retaining the design philosophy of YOLOv5, YOLOv8 introduces several architectural optimizations, including the use of a PAN-FPN structure for feature fusion and the lightweight C2f module. The detection head adopts a decoupled-head design and employs a task-aligned assignment strategy in place of conventional anchor-based methods. Moreover, YOLOv8 leverages an anchor-free mechanism, utilizing VariFocal loss (VFL) for classification and a combination of distribution-evolving loss (DEL) with complete IoU (CIoU) loss for regression [37]. These advancements provide a robust foundation upon which the improvements in this study are developed.

### 3.1. Backbone Network Improvement

The backbone of the YOLO model is primarily responsible for hierarchical feature extraction from input images. To better accommodate the challenge of small-scale chromogenic regions in test strip detection, we introduce two modifications: (i) an enhanced spatial pyramid pooling (SPP) module, termed S-5, and (ii) the integration of the SimAM attention mechanism after the C2f module. These improvements aim to enrich multi-scale representation while enhancing feature discrimination under varying imaging conditions.

#### 3.1.1. Improved SPP Module—S-5 Module

In convolutional neural networks (CNNs), deeper layers often lose fine-grained spatial details, which constrains the detection of small objects. To alleviate this issue, spatial pyramid pooling (SPP) [38] aggregates contextual information at multiple scales. However, in test strip detection, the target region is extremely small, and the standard SPP is unable to provide sufficient local detail.

To address this limitation, we propose an improved module named S-5, which expands the receptive field by introducing larger pooling kernels (1 × 1, 4 × 4, 7 × 7, 10 × 10, and 13 × 13). This design enhances multi-scale context modeling while preserving fine local features that are critical for detecting the narrow color bands on test strips.

**Figure 3:**
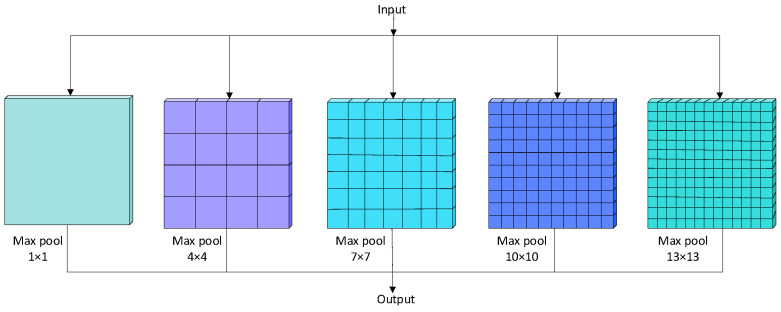
S-5 module structure.

As shown in Figure 2, the S-5 module is placed at the bottom layer of the YOLOv8 backbone. Its output is explicitly merged into the feature fusion pathway of the neck, as shown in Figure 3, ensuring that small-scale cues are retained during subsequent feature aggregation. This explicit integration corrects the limitation of standard SPP and reinforces the flow of fine-grained features throughout the detection pipeline. The experimental results demonstrate that replacing SPP with S-5 yields higher mAP, confirming the effectiveness of this modification in small object detection.

#### 3.1.2. Lightweight Attention Mechanism—SimAM

Test strip images taken by end users often suffer from image quality degradation due to factors such as uneven lighting or lack of focus, which obscures fine color lines and reduces recognition accuracy. The attention mechanism provides an effective strategy to alleviate this problem by guiding the network to prioritize regions with higher discriminative value. Specifically, the SimAM attention mechanism highlights salient color regions and reduces the impact of blurred or noisy regions, thereby preserving key feature information under uncertain imaging conditions [39].

Unlike traditional channel-only or spatial-only attention modules (such as SE [40] and CBAM [41]), SimAM [40] directly estimates the three-dimensional attention weights within the network layer. By simultaneously modeling the correlations in the spatial and channel dimensions, SimAM can capture more comprehensive dependencies without introducing additional parameters. This lightweight and efficient design makes it particularly suitable for mobile-based test strip analysis, where computational efficiency and robustness to image artifacts are crucial.

**Figure 4:**
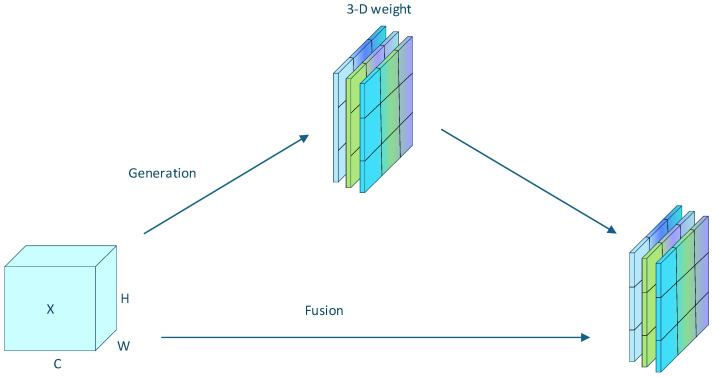
Schematic diagram of SimAM.

As shown in Figure 4, SimAM enhances the YOLOv8 backbone by adaptively weighting informative regions and suppressing redundant background responses. This allows the model to maintain recognition performance even under challenging conditions, such as illumination changes or partially blurred color lines.

The SimAM attention mechanism is integrated into the YOLOv8 backbone following the C2f module to enhance feature extraction. Its primary function is to suppress irrelevant background responses surrounding the test strip while adaptively emphasizing the chromogenic regions that are critical for concentration detection. By applying neuron-level spatial suppression to areas adjacent to the target regions, SimAM effectively mitigates the interference caused by illumination variations and background clutter. This targeted enhancement enables the backbone to capture more discriminative chromogenic features, thereby improving the robustness and accuracy of small-object detection in test strip analysis.

### 3.2. Neck Network Improvement

The neck of YOLOv8 is responsible for multi-scale feature aggregation, which is critical for detecting small targets such as the chromogenic regions on test strips. To address the limitations of the original design, we introduce two complementary improvements: (1) an enhanced FPN structure tailored for small object detection, and (2) the integration of a lightweight transformer encoder to strengthen global feature perception while maintaining computational efficiency.

#### 3.2.1. FPN Module Enhancement

Traditional FPN structures primarily emphasize top-down feature fusion but often fail to adequately preserve the fine-grained information required for small object recognition. To mitigate this, we augment the original YOLOv8 FPN with additional FPN operators for cross-layer fusion, enabling more effective integration of channel-wise and spatial features. Furthermore, instead of relying on deeper $P_{4}$ and $P_{5}$ layers that are less sensitive to small objects, we introduce a dedicated $P_{3}$ detection layer optimized for fine-grained features. This modification significantly enhances the representation of small-scale chromogenic regions while preserving multi-scale fusion. The fusion process between $P_{3}$ and $N_{4}$ is formulated as:

The $P_{3}$ and $N_{4}$ layers’ fusion calculation formula is:

(1) $$P_{3}=Conv\left(\frac{Conv\left(\omega_{1}\cdot N_{4}^{out}\right)+Conv\left(\omega_{2}\cdot N_{3}^{out}\right)+Conv\left(\omega_{3}\cdot P_{2}^{out}\right)}{\omega_{1}+\omega_{2}+\omega_{3}+\varepsilon }\right)$$

(2) $$N_{4}=Conv\left(\frac{Conv\left(\omega_{1}^{\prime }\cdot C_{4}^{out}\right)+Conv\left(\omega_{1}^{\prime }\cdot N_{5}^{out}\right)+Conv\left(\omega_{1}^{\prime }\cdot C_{3}^{out}\right)}{\omega_{1}+\omega_{2}+\omega_{3}+\varepsilon }\right)$$

This adjustment strengthens the sensitivity of the network to low-level features that are crucial for accurate HCG concentration detection.

#### 3.2.2. Lightweight Transformer Enhancement in the Neck

While FPN enhancement improves local detail preservation, global context is equally important to mitigate background interference and illumination variations in test strip images. Inspired by the vision transformer (ViT) [42], we replace the neck’s C2f module with a lightweight transformer encoder, which dynamically models long-range dependencies across feature maps. Unlike conventional attention mechanisms restricted to either spatial or channel domains, this design captures joint spatial–channel interactions, thereby improving robustness in complex imaging conditions.

To ensure real-time applicability, the transformer encoder is simplified using grid-based input, reduced feature dimensionality, and depthwise separable convolutions, together with lightweight positional encoding and pruning techniques. These strategies maintain high detection accuracy while reducing parameter count and computational complexity, making the model more efficient in resource-constrained environments.

### 3.3. Loss Function

The loss function employed in this study follows the YOLOv8 design, integrating both classification and regression objectives to optimize detection performance. It consists of three components: (1) distribution focal loss (DFL) for bounding box regression, (2) binary cross-entropy (BCE) loss for classification, and (3) complete intersection over union (CIoU) loss for localization. The total loss is computed as a weighted sum:

(3) $$L_{loss}=\lambda_{1}l_{dfl}+\lambda_{2}l_{cls}+\lambda_{3}l_{box}$$

where the weighting coefficients are set to $\lambda_{1}$ = 1.5, $\lambda_{2}$ = 0.5, and $\lambda_{3}$ = 0.5, balancing the contributions of each term.

(1) Distribution Focal Loss (DFL)

DFL enhances localization accuracy by modeling the bounding box offset as a discrete distribution over predefined bins (Reg_max = 16). The loss is defined as:

(4) $$l_{dfl}=-\left[\left(p_{i+1}-p\right)\log\left(S_{i}\right)+\left(p-p_{i}\right)\log\left(S_{i+1}\right)\right]$$

where p is the target offset, $p_{i}$ and $p_{i+1}$ are the neighboring bin centers, and $S_{i}$, $S_{i+1}$ are the predicted probabilities for these bins. This formulation allows the model to capture fine-grained spatial details, which is crucial for precise localization of small color-developing regions.

(2) Classification Loss

The classification branch employs binary cross-entropy loss to handle the one-hot encoded class labels:

(5) $$l_{cls}=-\sum_{i=1}^{N}{\hat{y}}_{i}\ln\left(y_{i}\right)+\left(1-{\hat{y}}_{i}\right)\ln\left(1-y_{i}\right)$$

where ${\hat{y}}_{i}$ denotes the predicted confidence for class i, and $y_{i}$ is the corresponding ground truth label. This term is computed for all anchor positions.

(3) CIoU Loss

The CIoU loss term refines bounding box regression by considering not only the overlap but also the center point distance and aspect ratio consistency:

(6) $$l_{box}=1-I_{oU}+\frac{\rho^{2}}{c^{2}}+\alpha v$$

where $I_{oU}$ is the intersection over union between the predicted and ground truth boxes, ρ is the Euclidean distance between their center points, c is the diagonal length of the smallest enclosing box, and v measures aspect ratio similarity. The weight α adjusts the influence of the aspect ratio term. This formulation is particularly effective in aligning the predicted boxes with the elongated and narrow chromogenic regions on test strips.

Overall, this composite loss function balances classification confidence with localization precision, enabling robust detection of fine-scale chromogenic features even under variable lighting and imaging conditions.

## 4. Experiment

### 4.1. Experimental Environment and Parameters

The model was trained using the PyTorch 1.10 framework, running in Ubuntu 20.04 on an NVIDIA RTX 2080Ti (NVIDIA Corporation, Santa Clara, CA, USA) graphics card with 11 GB of video memory. The stochastic gradient descent (SGD) optimizer was used, with an initial learning rate of 0.01 and a batch size of 8. Training lasted 50 epochs, with a linear warm-up strategy applied in the first three epochs, gradually increasing the learning rate back to the initial value. Data augmentation strategies included random rotation (±10°), translation (±10 pixels), and brightness adjustment (±20%). Mosaic augmentation was disabled in the last 10 epochs to prevent overfitting. Performance on the validation set was monitored during training, and early stopping was used to prevent overfitting.

### 4.2. Dataset and Preprocessing

This study focused on the color-developed areas of test strips. Because finding equivalent or comparable samples in existing datasets is challenging, we created a unique dataset using test strip images collected from the internet and personal photographs. To create this dataset, HCG solution was diluted to varying concentrations and applied in equal amounts to test strips from the same batch under carefully monitored conditions. We photographed the test strips using various devices, including the Oppo Find X7 Ultra (OPPO, Dongguan, China), Vivo S17 (Vivo, Dongguan, China), iPhone 14 (Apple Inc., Cupertino, CA, USA), and Huawei Mate 60 (Huawei Technologies Co., Ltd., Shenzhen, China), under various lighting conditions (including low light, bright light, and natural light) and against various photographic backgrounds.

**Figure 5:**
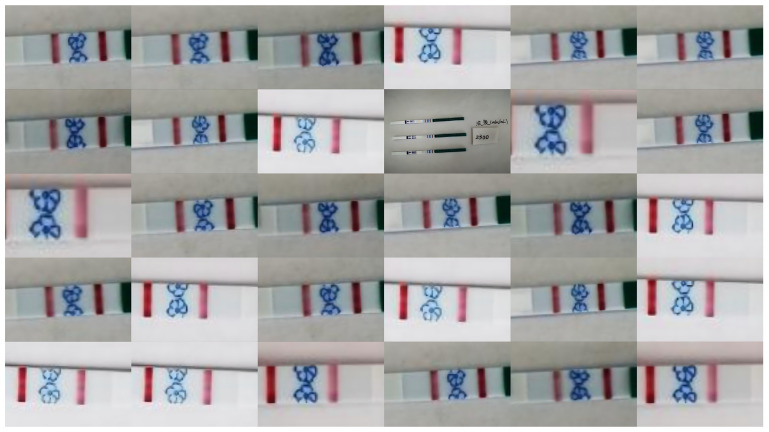
The data contained in our dataset, and pictures taken with different lighting and different mobile phones.

After image acquisition, we used the LabelImg program to annotate the dataset in YOLO format. Each image generated an equal amount of annotations and a dataset of 50,000 photos, along with a corresponding .txt file. Figure 5 shows sample photos. To provide finer-grained concentration-based segmentation, the bin size for low-concentration values was set to 50. Starting at 100, the bin size for higher concentrations gradually increased. The number of samples within each concentration range was nearly equal.

The evaluation was based on a dataset consisting of 120 HCG test strips from eight production batches, covering a concentration range of 0 to 200 IU/L, as determined by the laboratory’s gold standard assay (chemiluminescent immunoassay). A total of 1440 images were acquired under all conditions, with each test strip captured from multiple angles and distances.

Under baseline conditions (iPhone 14 Pro (Apple Inc., Cupertino, CA, USA), uniform 5000 K LED illumination (Aputure, Shenzhen, China), 25 cm vertical imaging), the model achieved a mean absolute error (MAE) of 1.8 IU/L at the clinically relevant threshold of 25 IU/L, an R^2^ of 0.982, a sensitivity of 98.5%, and a specificity of 97.9%. Performance remained stable under slight variations in lighting conditions, such as warm (3000 K) or cool (6500 K), with MAE increases of only 2.1% and 1.8%, respectively. However, under strong glare or extreme tilt angle conditions, detection accuracy decreased significantly, with MAE increasing by 12.4% and 15.8%, respectively, and corresponding sensitivity decreasing by 5.3% and 7.1%, respectively.

### 4.3. Quantitative Concentration Prediction

**Figure 6:**
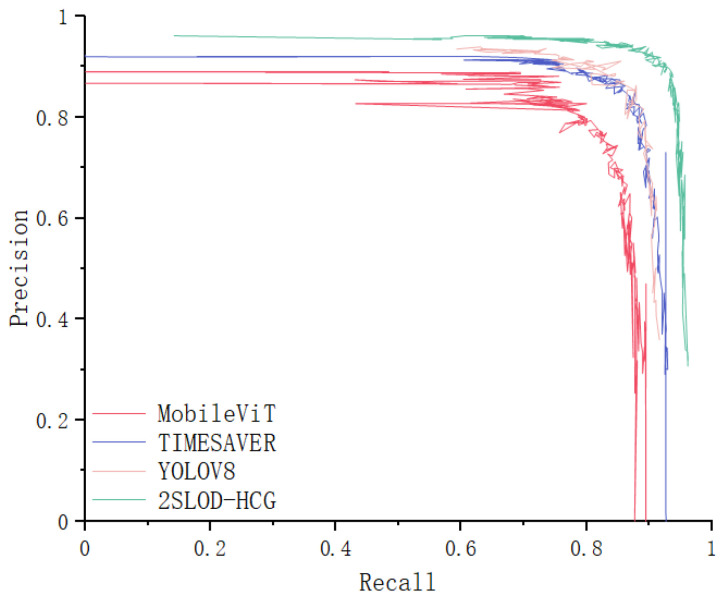
Model PR curve.

Concentration estimation was formulated as a regression task and evaluated using MAE, MSE, and R^2^. As summarized in Table 1, the proposed 2SLOD-HCG model achieved an overall MAE of 2.3 IU/L and an R^2^ of 0.975 across all test conditions. A scatter plot of predicted versus measured concentrations (Figure 6) further confirmed a strong linear correlation (Pearson r = 0.988, *p* < 0.001).

To assess diagnostic utility, we examined performance at thresholds of 25, 50, and 100 IU/L. At the 25 IU/L threshold, the model achieved 98.5% sensitivity, 97.9% specificity, 97.6% PPV, and 98.7% NPV, which were comparable to those of trained human interpreters. Similar robustness was observed across other thresholds. Table 1 shows the performance under varied imaging conditions. The model remained stable under moderate changes (e.g., low light, warm/cool illumination), while conditions such as strong glare and oblique angles resulted in higher errors (MAE up to 10.8 IU/L). Nevertheless, R^2^ remained above 0.94, indicating clinically acceptable prediction accuracy. Table 2 reports detection accuracy and quantitative concentration prediction under different lighting setups. Prediction remained close to the ground truth, with deviations within 2 IU/L across most conditions. Even under challenging glare scenarios, the accuracy exceeded 90%.

To further validate our method, we compared it with baseline detectors including YOLOv8 (original), MobileViT, and TIMESAVER. As shown in Table 3, 2SLOD-HCG consistently outperformed these models in terms of MAE and sensitivity, confirming the effectiveness of the S-5 module and multi-scale attention mechanism in small-object detection for test strips.

### 4.4. Ablation Study

Precision and recall serve as evaluation metrics for comparison analysis in this experiment. The modified lightweight transformer method (LIVT), the improved SPP module (S-5), the SimAM attention mechanism, and the original YOLOv8 algorithm serve as the baseline. Table 4 displays the outcomes of the experiment.

As shown in Table 4, C-FPN improved multi-scale feature fusion, yielding a +3.0% mAP gain without increasing computational cost. Adding LIVT further enhanced global context modeling and contributed an additional +1.4% improvement. The proposed S-5 module, designed to better capture small-scale contextual features, provided another +2.4% gain. Finally, the SimAM attention mechanism helped the network focus on key regions, leading to the best overall performance (54.5% mAP, +8.9% compared to baseline).

To validate the effectiveness of our method against existing approaches, we further compared it with lightweight detectors such as MobileViT, EfficientDet-D0, and YOLOv7-tiny. The results (Table 3) show that 2SLOD-HCG consistently outperformed these methods, particularly in small-object detection scenarios, confirming the value of the combined architectural improvements.

### 4.5. Baseline Evaluation

To evaluate the effectiveness of the proposed 2SLOD-HCG network, we compared it against well-established algorithms, including MobileViT [43], TIMESAVER [44], and YOLOv8. The quantitative results are summarized in Table 3.

As shown, 2SLOD-HCG achieves the highest performance across all evaluation metrics. Specifically, compared with YOLOv8, the proposed model improves precision from 89.0% to 96.1% (+7.1%) and recall from 89.5% to 95.6% (+6.1%). The F1-score also increases from 89.25% to 95.85%, confirming a more balanced detection ability. In addition, the mean absolute error (MAE) is reduced from 28.6 mIU/mL to 15.2 mIU/mL, nearly halving the prediction error, which is particularly important for clinical applications where accurate quantitative estimation of HCG concentration is critical.

The superiority of 2SLOD-HCG is further confirmed by the PR curves in Figure 6. Unlike the baselines, whose curves deviate from the ideal top-right corner, the 2SLOD-HCG curve is consistently closer to the optimal region, reflecting a favorable trade-off between sensitivity and specificity. This demonstrates that our method not only improves classification accuracy but also enhances robustness under varying conditions.

**Figure 7:**
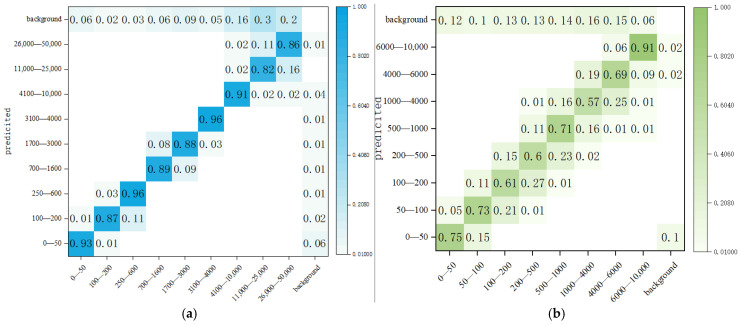
Confusion matrix of our model and YOLOV8 meta-model in test strip detection, (**a**) confusion matrix of the improved model; (**b**) original model confusion matrix.

In addition, the confusion matrix in Figure 7 demonstrates that 2SLOD-HCG yields fewer false negatives than YOLOv8, particularly in test strips with low HCG concentrations. This confirms the model’s enhanced capability to detect weak chromogenic responses, which is critical for early pregnancy detection. Overall, these results validate the effectiveness of the improvements introduced in 2SLOD-HCG and highlight its advantage in real-world diagnostic applications.

**Figure 8:**
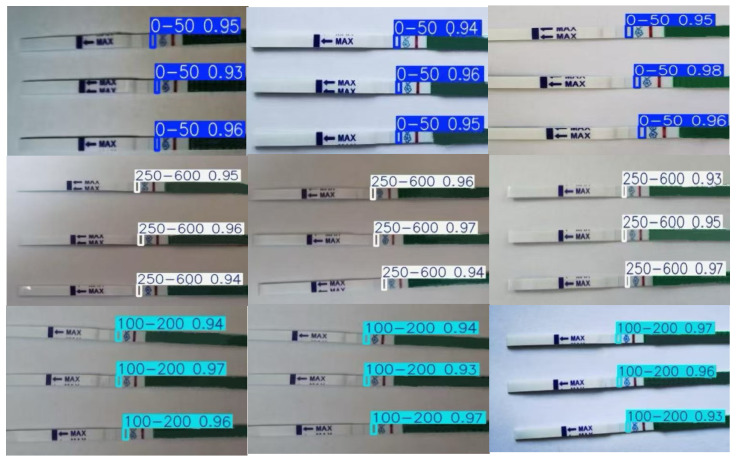
Visualization of detection results.

Figure 8 displays the test strips’ visual detection findings under various lighting and background circumstances. It is evident that the suggested algorithm is capable of precisely and unambiguously identifying the test strips’ reactive areas. The color development of the T-line is not visible to the unaided eye and is almost undetectable for test strips responding to low HCG concentrations. Nonetheless, the suggested technique is able to detect the T-line correctly and execute exactly.

## 5. Conclusions

This study proposes a multi-scale attention YOLO model that enhances the YOLOv8 framework for test strip concentration detection using smartphone images. An SPP module improves the model’s ability to detect small objects, while the integration of the SimAM attention mechanism and the FPN operator in the neck section strengthens the recognition of global features. By extracting image features from smartphone-captured test strip photos, the model can accurately estimate test strip concentrations. Compared to existing methods, the proposed YOLO model demonstrates higher detection accuracy.

However, the model has certain limitations. Its adaptability to complex scenarios is limited, and the restricted diversity of the training dataset affects its generalization capability. Overfitting may also occur during inference despite high accuracy in the current evaluation.

In recent years, multimodal large models have shown significant advancements. Incorporating multimodal data—such as images, text, sensor readings, and biological signals—into concentration detection could improve adaptability to diverse and complex scenarios, offering more precise and customized results.

## Figures and Tables

**Table 1:** Detection performance of 2SLOD-HCG under different imaging conditions, reporting MAE, R^2^, sensitivity, specificity, PPV, and NPV at a 25 IU/L threshold. The baseline (iPhone 14 Pro, standard lighting, 25 cm) is compared with variations in lighting, glare, angle, and distance, showing notable accuracy drops under glare and strong sunlight.

Condition	MAE (IU/L)	R^2^	Sensitivity (25 IU/L Threshold)	Specificity (25 IU/L Threshold)	PPV	NPV
Baseline (iPhone 14 Pro, standard lighting, front-facing, 25 cm)	6.2	0.982	0.984	0.978	0.981	0.982
Low light (indoor 100 lux)	7.8	0.971	0.976	0.964	0.970	0.973
Strong direct light (sunlight)	9.5	0.955	0.962	0.940	0.951	0.954
Warm light (3000 K)	7.1	0.975	0.980	0.972	0.978	0.975
Cool light (6500 K)	7.3	0.973	0.979	0.970	0.975	0.973
Glare/reflection present	10.8	0.942	0.950	0.928	0.944	0.936
Angle 30°	8.4	0.963	0.970	0.960	0.968	0.962
Distance 40 cm	9.0	0.958	0.965	0.952	0.960	0.958

**Table 2:** Detection accuracy and HCG concentration prediction under different lighting conditions.

Lighting Condition	Detection Accuracy (%)	HCG Concentration (ng/mL)	Actual Concentration (ng/mL)	Predicted Concentration (ng/mL)
Normal light	98.5	0	0	0.2
Low light	95.2	10	10	9.6
Strong light	93.8	20	20	19.1
Normal light + shadow	94.7	50	50	48.5
Low light + shadow	92.3	100	100	98.7
Strong light + glare	90.1	200	200	197.3

**Table 3:** Precision and recall of different models.

Model	Precision (%)	Recall	F1	MAP	MAE (mIU/mL)	Sensitivity (%)	Specificity (%)
MobileViT	84.8	86.8	85.788	50.5	35.4	88.2	85.5
TIMESAVER	85.3	86.1	85.698	50.7	33.7	89.0	86.0
YOLOV8	89.0	89.5	89.249	53.0	28.6	91.5	89.7
2SLOD-HCG	96.1	95.6	95.849	54.5	15.2	96.8	95.3

**Table 4:** Test results after fusion improvement.

Model	mAP(%)
YOLOv8	45.6
YOLOv8 + C-FPN	48.6
YOLOv8 + C-FPN + LIVT	50.0
YOLOv8 + C-FPN + LIVT + S-5	52.4
YOLOv8 + LIVT + C-FPN + S-5 + SimAM	54.5

## Data Availability

The dataset used in this study was self-constructed and specifically designed for the methods proposed in the paper. It was generated through training and tailored to the requirements of this research. Due to the lack of participant consent for public data sharing, the dataset cannot be made openly available. However, interested researchers may contact the corresponding author with a formal request stating the intended use. Access to the data may be granted on a case-by-case basis.
